# Hospital resource utilization in a national cohort of functionally single ventricle patients undergoing surgical treatment

**DOI:** 10.1016/j.xjon.2023.03.014

**Published:** 2023-04-08

**Authors:** Dan-Mihai Dorobantu, Qi Huang, Ferran Espuny Pujol, Katherine L. Brown, Rodney C. Franklin, Maria Pufulete, Deborah A. Lawlor, Sonya Crowe, Christina Pagel, Serban C. Stoica

**Affiliations:** a Children's Health and Exercise Research Centre, University of Exeter, Exeter, United Kingdom; b Population Health Sciences, University of Bristol, Bristol, United Kingdom; c University Hospitals Bristol and Weston National Health Service Foundation Trust, Bristol, United Kingdom; d Clinical Operational Research Unit, Department of Mathematics, University College London, London, United Kingdom; e Cardiac and Critical Care Division, Great Ormond Street Hospital National Health Service Foundation Trust, London, United Kingdom; f Department of Paediatric Cardiology, Royal Brompton and Harefield National Health Service Foundation Trust, London, United Kingdom; g Bristol Heart Institute, University of Bristol, Bristol, United Kingdom; h Medical Research Council Integrative Epidemiology Unit at the University of Bristol, Bristol, United Kingdom; i Bristol National Institute for Health Research Biomedical Research Centre, Bristol, United Kingdom

**Keywords:** Fontan, hospital length of stay, hospital resources, hypoplastic left heart syndrome, Norwood, registry, single ventricle

## Abstract

**Objective:**

The study objective was to provide a detailed overview of health resource use from birth to 18 years old for patients with functionally single ventricles and identify associated risk factors.

**Methods:**

All patients with functionally single ventricles treated between 2000 and 2017 in England and Wales were linked to hospital and outpatient records using data from the Linking AUdit and National datasets in Congenital HEart Services project. Hospital stay was described in yearly age intervals, and associated risk factors were explored using quantile regression.

**Results:**

A total of 3037 patients with functionally single ventricles were included, 1409 (46.3%) undergoing a Fontan procedure. During the first year of life, the median days spent in hospital was 60 (interquartile range, 37-102), mostly inpatient days, mirroring a mortality of 22.8%. This decreases to between 2 and 9 in-hospital days/year afterward. Between 2 and 18 years, most hospital days were outpatient, with a median of 1 to 5 days/year. Lower age at the first procedure, hypoplastic left heart syndrome/mitral atresia, unbalanced atrioventricular septal defect, preterm birth, congenital/acquired comorbidities, additional cardiac risk factors, and severity of illness markers were associated with fewer days at home and more intensive care unit days in the first year of life. Only markers of early severe illness were associated with fewer days at home in the first 6 months after the Fontan procedure.

**Conclusions:**

Hospital resource use in functionally single ventricle cases is not uniform, decreasing 10-fold during adolescence compared with the first year of life. There are subsets of patients with worse outcomes during their first year of life or with persistently high hospital use throughout their childhood, which could be the target of future research.

## Graphical abstract

**Figure:**
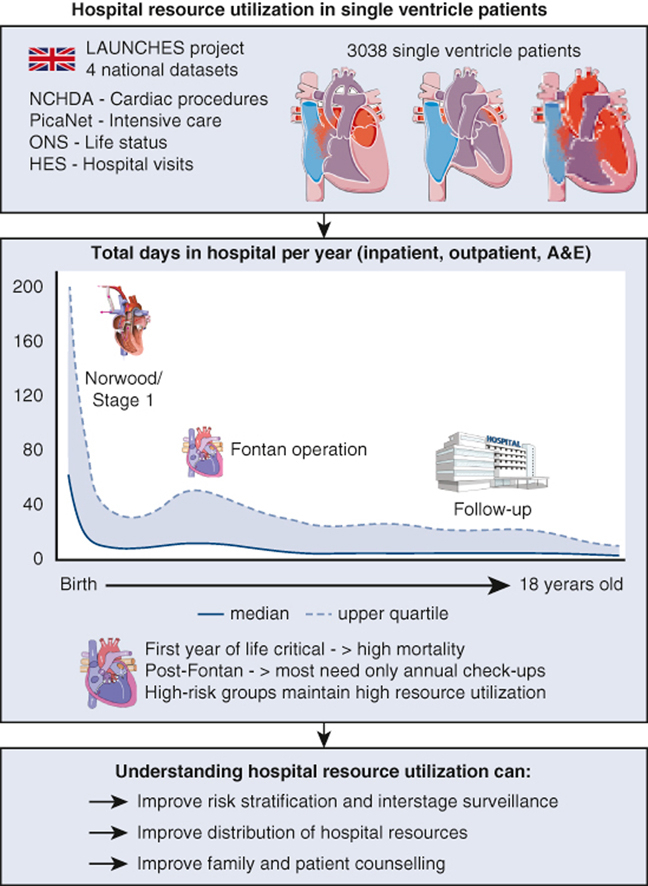

**Figure:**
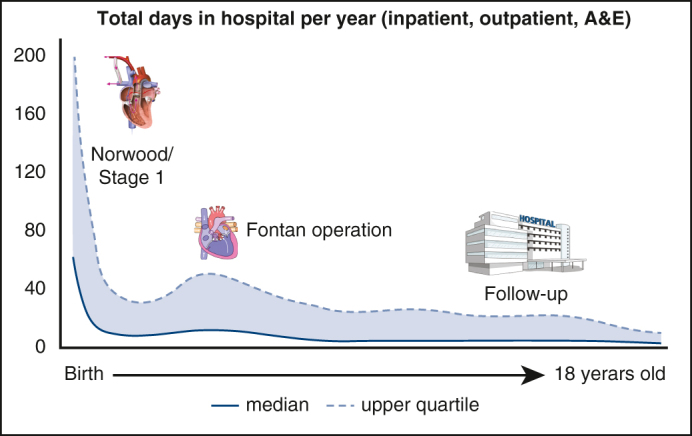
Total hospital resource use in f-SV from birth to 18 years old.

**Central Message**

After resource-intensive first years of life, most adolescents with Fontan circulation will only need yearly outpatient visits, with a minority maintaining high hospital resource use.

**Perspective**

This study describes in new detail the time spent in hospital and outpatient episodes, from birth to adult care in all patients with single ventricles receiving at least 1 intervention. These results can improve how single ventricle–associated healthcare resources are distributed by policy makers across age groups, counseling of families and patients, and identifying high-risk populations.

Functionally single ventricle (f-SV) encompasses a wide variety of congenital heart diseases (CHDs), where a biventricular circulation cannot be established, currently treated with a 3-staged approach.^1^ Advances in surgical techniques, critical care, and follow-up improved the outcomes, which still remain less favorable than for other complex CHD.^2^, ^3^, ^4^, ^5^, ^6^

Treatment in f-SV is highly resource intensive through the time spent in hospital and financial and logistical costs.^7^, ^8^, ^9^, ^10^, ^11^ Current data focus on each treatment stage^12^, ^13^, ^14^, ^15^, ^16^, ^17^ and certain aspects of care, such as total hospital costs,^10^, ^11^, ^15^ noncardiac hospitalizations,^7^, ^18^ and costs associated with poor outcome.^19^ Longitudinal studies, such as the Philadelphia fetus-to-Fontan study,^8^ the Australia/New Zealand registry,^20^ or the Utah statewide analysis,^21^ provide insights into resource use associated with f-SV care, but limited to recipients of the Fontan operation, or without discriminating between inpatient or outpatient stay. A complete overview of hospital stays in f-SV including cardiac and noncardiac, inpatient and outpatient, from birth to adult care is not available. This gap in knowledge limits clinicians' ability to evaluate optimal management, policy makers' ability to allocate resources, and families' opportunities to adjust and plan to what their expected real-life challenges would be, despite this latter point being a major source of anxiety for parents.^22^

As part of the Linking AUdit and National datasets in Congenital HEart Services for Quality Improvement (LAUCHES QI) project,^23^ the current study aims to (1) evaluate hospital resource use in f-SV treatment, from birth to late adolescence; (2) investigate factors associated with fewer days spent at home and more days spent in the intensive care unit (ICU) during the first year of life of infants with f-SV; and (3) report hospital resource use after the Fontan procedure, and factors associated with postoperative ICU stay, and fewer days at home within 6 months of the Fontan operation.

## Patients and Methods

### LAUNCHES QI: Linking UK National Datasets on Congenital Heart Disease Care

The LAUNCHES QI project links several national datasets from the United Kingdom, evaluating outcomes and hospital resource use related to CHD care, with full linkage of all datasets covering England and Wales. These include the National Congenital Heart Disease Audit (NCHDA), which collects procedure based information from all CHD centers (April 2000-March 2017); the pediatric intensive care audit network (PICANet) for admissions to pediatric ICUs (2001 to March 2017); death registrations from the Office for National Statistics (ONS) (up to February 2022); and hospital episode statistics (HES), which contain routine administrative data on inpatient, outpatient, and emergency care at hospitals in England (inpatient April 2000 to March 2017, outpatient April 2003 to March 2017, accidents and emergency department [A&E] April 2007 to March 2017).^23^ The LAUNCHES project received ethical approval from the Health Research Authority (reference: IRAS 246796) and the Confidentiality Advisory Group (reference: 18/CAG/0180) in accordance with the Declaration of Helsinki.

### Clinical Data Collection, Classification, and Management

**Figure 1:**
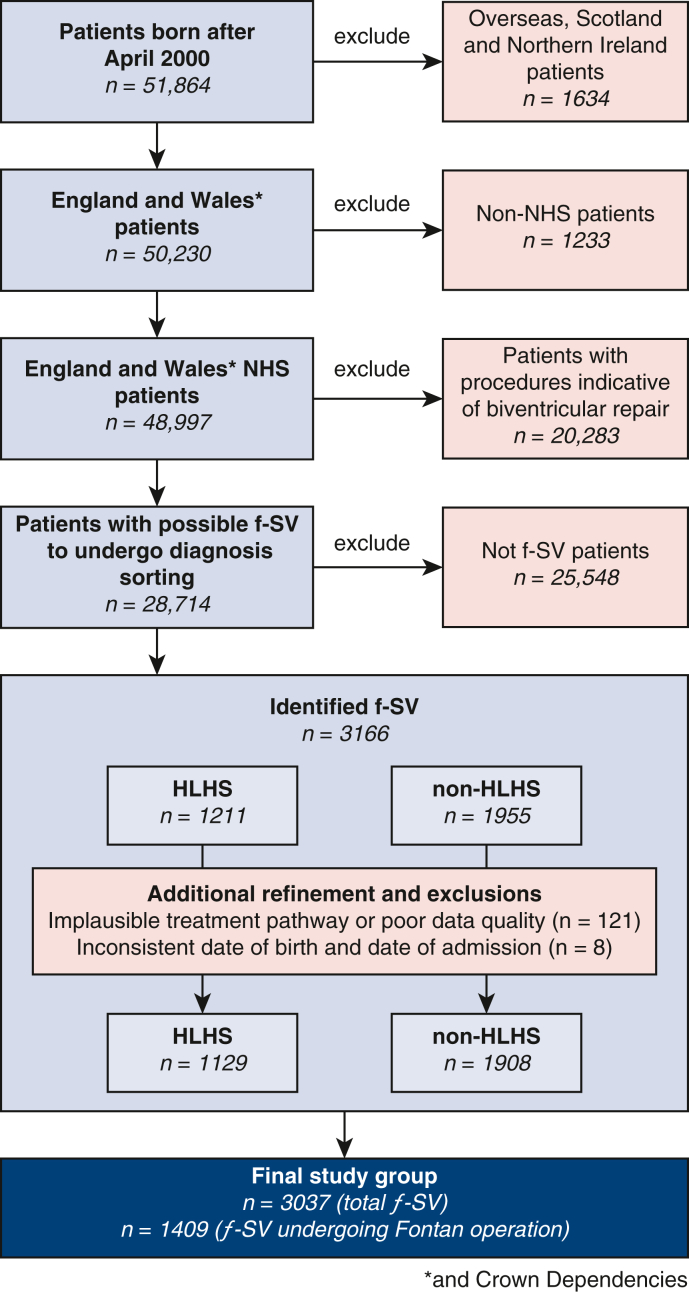
Flowchart of f-SV study population inclusion and exclusion steps and classifications. Identification of patients with and without HLHS, and classification into diagnosis types were done using an algorithm described previously^2^^,^^6^ and further in the Appendix E1. *NHS*, National Health System; *f-SV*, functionally single ventricle; *HLHS*, hypoplastic left heart syndrome.

The patient inclusion and exclusion steps are summarized in Figure 1. All clinical data were organized into patient care episodes or “spells,” containing patient events separated by no more than 24 hours,^23^ classified as cardiac or noncardiac. Survival data for a cohort encompassing the currently analyzed population were previously reported.^5^

Cardiac diagnosis and procedure codes^24^ were used to identify patients with f-SV: First, those undergoing biventricular repair were excluded;^2^ next, a stepwise algorithm was used to identify hypoplastic left heart syndrome (HLHS)^6^ and non-HLHS subtypes.^5^

The following patient/procedure data were extracted from the available datasets (detailed in Appendix E1 and Table E1, Table E2, Table E3, Table E4): age and weight, gender, anatomic diagnosis within 8 defined subgroups,^2^, ^6^ preoperative risk factors,^25^ year of procedures, type of stage 1 procedure,^2^ stage 2/stage 3 status, number of off-pathway procedures performed that are not part of the Fontan pathway, such as revisions of the arterial shunts, conduit reinterventions or recoarctation treatment,^2^, ^6^ total cardiopulmonary bypass (CPB) time in the first year of life (as a surrogate for number and complexity of cumulated surgical procedures), and Fontan operation crossclamp duration.

Each dataset has a different start date and end date, and data collection rules vary by time, such as preoperative risk factors being collected systematically after 2009. This has led to each analysis being performed for periods when all required datasets overlapped, mentioned at each respective step. For hospital resource use, left truncation occurs where data were available only from a given age onward. Patients presenting in the lower age limit of age intervals who die or are censored before the upper age limit are considered to not have complete hospital use data for that interval (right censoring).

In addition, PICANet uses an internal censoring mechanism, where entries of patients aged more than 18 years can no longer be identified, so those born before 2002 were not linked. Details on data collection, classification, and management are available in the Appendix E1 and Table E1, Table E2, Table E3, Table E4.

### Outcomes

#### Hospital resource use outcomes

Hospital resource use included (1) days in hospital: total (inpatient, outpatient and A&E), inpatient, outpatient, and ICU days during the first year of life (1-month age intervals; (2) days in hospital: total (inpatient, outpatient and A&E), inpatient, outpatient, and ICU days from 0 to 18 years (1-year age intervals); (3) days at home during the first year of life; (4) inpatient care episode (spell) length and ICU days post-Fontan procedure; and (5) inpatient days and days at home in the first 6 months post-Fontan procedure. Care episode length and ICU length of less than 1 day are counted as 1 day.

#### Vital status

Patient life status was provided at the point of hospital discharge by the NCHDA for each cardiac procedure and at date of extract by the Office of National Statistics (national death registry data). Any patients with missing ONS life status were censored at their last known live discharge age.

### Statistical Analyses

Frequencies are reported as numbers and proportions, and continuous variables as median with interquartile range (IQR) and minimum-maximum range, as well as means in Table E10, Table E11, Table E12, Table E5, Table E6, Table E7, Table E8, Table E9. Any missing values affecting the denominator or leading to exclusions from analyses are detailed where relevant and were not imputed because of the high level of data completeness.

### Hospital Resource Use (First Year of Life and 0-18 Years)

Median (IQR) days in hospital/ICU were counted within each age interval. Intervals of 1 month were used for the first year of life, and 1-year age intervals were used for 2 to 18 years. The numbers of patients who died or were right-censored within each interval are reported (Table E10, Table E11, Table E12, Table E5, Table E6, Table E7, Table E8, Table E9).

### Factors Associated With Days at Home and Intensive Care Unit Time Analysis

To investigate factors associated with hospital resource use, 4 outcomes of interest were used: days at home and days in ICU during first year of life, postoperative ICU days during the Fontan episode (spell) of care, and days at home within 6 months post-Fontan. Patients who died before year 1 or within 6 months of a Fontan procedure were assigned a value of zero days at home as the worst outcome.^26^, ^27^ Patients censored in HES/PICANet during the first year of life (or within 6 months of Fontan procedure) were excluded from the respective days at home/days in ICU analyses.

Quantile regression was carried out to explore factors associated with the median of each outcome of interest, because the data are highly skewed.^28^ Effects are presented as univariable and adjusted coefficients and 95% confidence intervals (CIs). Clustered standard errors were computed for all models to allow for intra-center correlation.^28^^,^^29^ Clinically relevant factors were selected as candidate explanatory variables in the models (detailed in Appendix E1).

All statistical analyses were performed using STATA/SE 17.0 (StataCorp LLC), and figures were created using R version 4.1.2 (R Foundation for Statistical Computing).

## Results

A total of 3037 patients with f-SV, HLHS, and other non-HLHS subtypes of f-SV undergoing their first cardiac procedure between April 2000 and March 2017 were included (Figure 1). Table 1 shows detailed demographic, clinical, and procedural data for the whole cohort (n = 3037) and the Fontan procedure subgroup (n = 1409, 46.3%).

**Table 1:** Demographic, clinical, and procedural data in all functional single ventricle patients (n = 3037) and those undergoing the Fontan operation (n = 1409)

Demographic and anthropometric	All patients	Fontan subgroup
Age at first procedure/Fontan (median, IQR)	6 (3-28) (d)∗	4.5 (3.7-5.7) (y)†
Weight, kg (median, IQR)‡	3.2 (2.9-3.8)∗	16 (14.4-18.5)†
Low weight <2.5 kg at first procedure, n (%)‡	293 (9.7)	103 (7.3)
Male, n (%)	1784 (58.7)	847 (60.1)
Anatomic subtype, n (%)		
HLHS	1129 (37.2)	404 (28.7)
Single ventricle with isomerism	222 (7.3)	90 (6.4)
Double inlet left ventricle	303 (10.0)	189 (13.4)
Tricuspid atresia	390 (12.8)	224 (15.9)
Non-HLHS mitral atresia	102 (3.4)	52 (3.7)
Unbalanced atrioventricular septal defect	223 (7.3)	79 (5.6)
Pulmonary atresia	168 (5.5)	95 (6.7)
All other single ventricle cases	500 (16.5)	276 (19.6)
Preoperative clinical risk factors, n (%)§		
Antenatal diagnosis‖	1270 (85.5)	364 (85.3)
Preterm birth	122 (8.2)	23 (5.4)
Congenital noncardiac comorbidity	296 (19.9)	65 (15.2)
Additional cardiac risk factors	120 (8.1)¶	15 (3.5)†
Acquired comorbidity	115 (7.8)∗	52 (12.2)† 48 (11.2)#
Severity of illness marker	302 (20.3)∗	6 (1.4)† 84 (19.7)#
Procedure related factors		
Stage 1 performed, n (%)	2648 (87.2)	1201 (85.2)
Stage 1 subtype, n (%)		
Only Norwood	1276 (42.0)	522 (37.1)
Only hybrid HLHS	89 (2.9)	11 (0.8)
Coarctation/interrupted arch repair	147 (4.8)	81 (5.8)
Securing pulmonary blood flow	785 (25.9)	413 (29.3)
Protecting the pulmonary vascular bed	337 (11.1)	173 (12.3)
Hybrid HLHS followed by Norwood	14 (0.5)	1 (0.1)
Stage 2 performed, n (%)	2184 (71.9)	1357 (96.3)
Off-pathway procedure, n (%)	1495 (49.2)	825 (58.6)
Procedure era, n (%)		
2000-2005	867 (28.5)∗	98 (7.0)†
2006-2010	1030 (33.9)∗	476 (33.8)†
2011-2016	1140 (37.5)∗	835 (59.2)†

*IQR*, Interquartile range; first procedure is always first cardiac procedure; *HLHS*, hypoplastic left heart syndrome.

∗ At first procedure.

† At Fontan procedure.

‡ Missing in n = 61 overall and n = 45 Fontan patients.

§ Reported n = 1485 overall and n = 427 Fontan patients (birth since 2009 or later).

‖ Missing in n = 6 overall and n = 1 Fontan patients.

¶ During first year of life.

# Before Fontan procedure.

### Hospital Resource Use by Age Intervals

There were 176,751 care episodes (spells) analyzed after exclusion of unattended outpatient episodes (n = 23,985) and those with age at admission anomalies (admission age later than age at death, n = 213). Of these, 45% were cardiac and the remainder were noncardiac or ambiguous. By admission type, 19.5% were inpatient and 75.5% were outpatient, whereas 5% were A&E attendance without admission. Of the noncardiac/ambiguous episodes with reported cause, the most common were anticoagulant service (8.3%), hematology (6.7%), respiratory conditions (5.2%), dietetics (2.7%), and dentistry (2.5%).

#### During the first year of life

**Figure 2:**
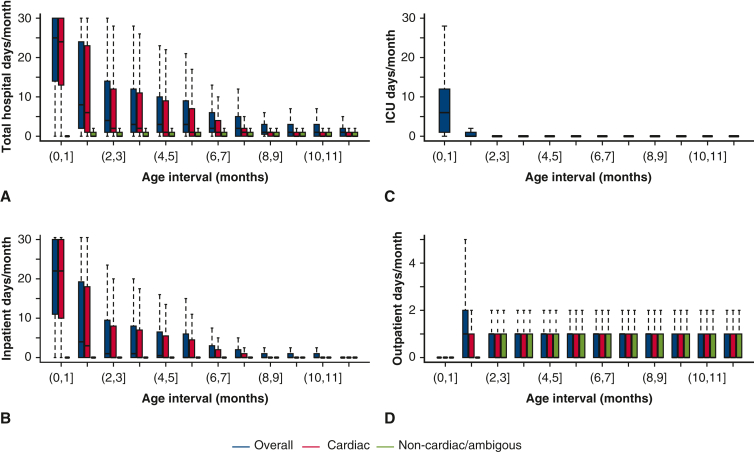
Hospital resource use in patients with f-SV from birth to 1 year of life. Reported as median number of days/month within 1-month age intervals. A, Total days spent in the hospital (inpatient, outpatient, and A&E without admission, cardiac, and noncardiac/ambiguous). B, Number of inpatient days (cardiac and noncardiac/ambiguous). C, Days in ICU. D, Number of outpatient days (cardiac and noncardiac/ambiguous). A to C show the median (*horizontal black line line*), IQR (*colored solid bars*), and 1.5× IQR (*dotted vertical lines*), whereas outliers outside these limits are not shown. Corresponding numerical values, including means, number of death, and censoring in each age intervals, are detailed in Table E5, Table E6, Table E7, Table E8. ICU data are available for patients who were born in 2002 and onward. Patients were included in each consecutive age interval if they had data (linked and available) and were alive or not censored in the lower age limit. *ICU*, Intensive care unit.

The median number of days in hospital (inpatient, outpatient, or A&E without admission) decreases from 25 days [IQR, 14-30] in the first month, to 8 days [IQR, 2-14] in the second month, and to 3 to 4 days between 3 and 6 months and 1 to 2 days between 7 and 12 months of age (Figure 2, *A*). Likewise, most inpatient and ICU days were during the first 2 months of life (Figure 2, *B* and *C*, respectively.) The outpatient visits in the first year had a median of at most 1 day/month (Figure 2, *D*). Median, IQR, range, and mean values, as well as the number of deaths and censoring in each age intervals for Figure 2 are provided in Table E5, Table E6, Table E7, Table E8. The majority of hospital days were for cardiac causes.

#### From birth to 18 years old

**Figure 3:**
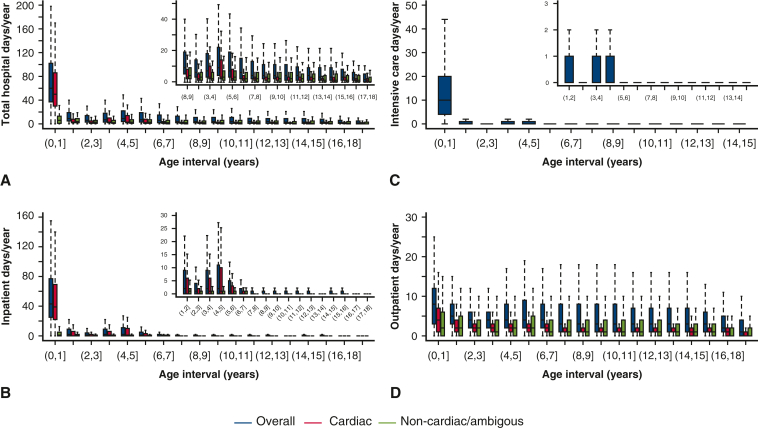
Hospital resource use in patients with single ventricle from birth to 18 years of life. Reported as median number of days/year, within 1-year age intervals. A, Total days spent in the hospital (inpatient, outpatient, and A&E without admission, cardiac and noncardiac/ambiguous). B, Number of inpatient days (cardiac and noncardiac/ambiguous). C, Days in ICU. D, Number of outpatient days (cardiac and noncardiac/ambiguous). A to D show the median (*horizontal black line line*), I (*colored solid bars bars*), and 1.5× IQR (*dotted vertical lines*). Inset panels show years 1 to 18 of life, with adjusted scale, after excluding the first year. Outliers outside these limits are not shown. Corresponding numerical values, including means, number of deaths, and censoring in each age intervals, are detailed in Table E10, Table E11, Table E12, Table E9. ICU data are available for patients who were born in 2002 and onward. Patients were included in each consecutive age interval if they had data (linked and available) and were alive or not censored in the lower age limit.

The overall median number of days spent in hospital (inpatient, outpatient, or A&E without admission) decreased from 60 days [IQR, 37-102] for the first year of life to fewer than 4 days/year for ages 7 to 18 years, with a small increase around 3 to 6 years (the Fontan operation age) (Figure 3, *A*). A similar pattern is seen in inpatient hospital days/year (from 43 to 0 days/year) (Figure 3, *B*) and days in ICU/year (from 10 to 0 days/year) (Figure 3, *C*), and to a lesser degree outpatient days/year (from 8 to 1-3 days/year) (Figure 3, *D*). Median, IQR, range, and mean values for Figure 3 are provided in Table E10, Table E11, Table E12, Table E9. A&E without admission amounts on average to less than 1 day/year per patient. Table E10, Table E11, Table E12, Table E9 also detail deaths within each 1-year follow-up intervals.

### Factors Associated With Hospital Resource Use During the First Year of Life

There were 693 (n = 22.8%) deaths during the first year of life, mostly in-hospital (n = 633/693, 91.3%). During the same period, an ICU admission was recorded in 2589 patients (95%) and extracorporeal membrane oxygenation (ECMO) in 122 patients (4.5%). The number of inpatient days, ICU days, and ECMO days during the first year of life by survivor status and stage are shown in Table 2. Nonsurvivors had fewer inpatient days on average (of them only 10% reached stage 2), but with longer ICU stays and more ECMO days. Most of the resource use was related to stage 1 and less so to stage 2 procedures. For patients undergoing at least 1 on-pump procedure, the median total CPB time during the first year of life was 2 hours (IQR, 1.4-2.6, range, 0.1-10.1).

**Table 2:** Hospital resource use (number of inpatients, intensive care unit and extracorporeal membrane oxygenation days) in the first year of life for functionally single ventricle patients, overall, in nonsurvivors, and in relation to stage 1 and stage 2 procedure episodes (spells)

No. of inpatient days∗				
	Mean	Median (IQR)	Range	No. of patients
Overall	62.6	43 (25-77)	(0-365)	2819
Nonsurvivors	55.3	34 (14-74)	(1-321)	576
Stage 1 related	37.2	22 (11-42)	(0-365)	2819
Stage 2 related	10.2	1 (0-11)	(0-305)	2819
No. of ICU d†				
Overall	18.2	10 (4-20)	(0-365)	2726
Nonsurvivors	23.7	14 (6-30)	(0-226)	611
Stage 1 related	13.6	7 (2-14)	(0-365)	2726
Stage 2 related	2.7	0 (0-2)	(0-212)	2726
No. of ECMOs d†				
Overall	1.0	0 (0-0)	(0-121)	2726
Nonsurvivors	2.3	0 (0-0)	(0-110)	611
Stage 1 related	0.8	0 (0-0)	(0-121)	2726
Stage 2 related	0.2	0 (0-0)	(0-78)	2726

*IQR*, Interquartile range; *ICU*, intensive care unit; *ECMO*, extracorporeal membrane oxygenation.

∗ n = 190 patients were not linked to HES and n = 28 patients have inpatient episodes (spells) in the first of their life without a discharge date, excluded.

† Only in patients born in April 2002 or later, due to PICANet censoring data of patients once they reach 18 y of age. Also excluded are n = 30 patients were not linked to PICANet for other reasons.

Factors associated with fewer days spent at home and more days spent in the ICU during the first year of life from the univariable and multivariable analyses are shown in Table 3.

**Table 3:** Factors associated with days spent at home and in the intensive care unit in the first year of life in functionally single ventricle patients

	Days at home (first year of life)		Days in ICU (first year of life)	
	Univariate coefficient (95% CI)	Adjusted coefficient (95% CI)	Univariate coefficient (95% CI)	Adjusted coefficient (95% CI)
Clinical subgroup				
HLHS	−80 (−128.4 to −31.6)∗	−54.7 (−92.7 to −16.7)∗	9 (5.1-12.9)†	7.8 (3.4-12.2)†
Single ventricle with isomerism	−11 (−26.3 to 4.3)	−4.1 (−17.7 to 9.6)	0 (−2.3 to 2.3)	0.1 (−2.5 to 2.6)
Double inlet left ventricle	7 (−21.5 to 35.5)	2.5 (−14.6 to 19.6)	−1 (−3.4 to 1.4)	−0.1 (−2.9 to 2.6)
Tricuspid atresia	Reference	Reference	Reference	Reference
Mitral atresia	−71 (−105.7 to −36.3)†	−55.4 (−96.5 to −14.3)∗	7 (2.0-11.9)∗	5.2 (−0.2 to 10.6)‡
Unbalanced atrioventricular septal defect	−81 (−138.4 to −23.6)∗	−65.1 (−132.2 to 2.0)‡	6.2 (0.4 to 12.1)∗	3.0 (−1.2 to 7.3)
Pulmonary atresia	−9 (−33.7 to 15.7)	−4.9 (−20.5 to 10.8)	1 (−1.7 to 3.7)	0.1 (−2.7 to 2.8)
All other single ventricle	5 (−16.4 to 26.4)	−2.1 (−16.2 to 11.9)	−2 (−4.2 to 0.2)‡	−1.2 (−4.2 to 1.8)
Male gender	4 (−9.4 to 17.4)	3.4 (−5.0 to 11.8)	1 (−1.4 to 3.4)	0.2 (−1.9 to 2.2)
Congenital comorbidity	−55 (−74.9 to −35.1)†	−31.5 (−46.4 to −16.7)†	3 (0.7 to 5.3)§	2.0 (−0.1 to 4.2)‡
Preterm birth	−75 (−95.3 to −54.7)†	−52.5 (−82.4 to −22.5)∗	4 (−2.0 to 10.0)	1.4 (−1.1 to 3.9)
Antenatal diagnosis	−13 (−20.8 to −5.2)∗	−11.7 (−23.0 to −0.4)§	−2 (−4.8 to 0.8)	0.2 (−2.0 to 2.3)
Additional cardiac risk (first year of life)	−100 (−134.7 to −65.2)†	−52.8 (−97.0 to −8.5)§	15 (9.7 to 20.3)†	9.6 (2.2 to 16.9)§
Acquired comorbidity (first procedure)	−112 (−177.0 to −47.0)∗	−74.0 (−114.4 to −33.5)†	10 (0.3 to 19.7)§	4.0 (−2.9 to 10.9)
Severity of illness marker (first procedure)	−56 (−80.2 to −31.8)†	−26.4 (−37.1 to −15.8)†	11 (6.7 to 15.3)†	7.0 (2.2 to 11.7)∗
Low weight <2.5 kg (first procedure)	−140 (−238.7 to −41.3)∗	−67.4 (−167.8 to 33.0)	12 (5.2 to 18.8)†	7.7 (3.4 to 12.1)∗
Age in mo (first procedure)	2.1 (1.7 to 2.5)†	1.4 (0.8 to 2.0)†	−0.5 (−0.7 to −0.2)†	−0.3 (−0.5 to −0.2)†

Quantile (at median) analysis performed for patients born in April 2009 or later (n = 1,485, exclusions below). N = 1 (weight missing) and n = 6 (antenatal diagnosis missing) excluded from all regression analysis; n = 44 excluded from days-at-home analysis (not linked to HES, censored in HES in first year of life or have inpatient episodes (spells) without discharge age in first year of life); n = 117 excluded from the days in ICU analysis (not linked to PICANet or censored in PICANet in first year of life). If no cardiac procedure in first year, related comorbidities marked as “no.” Coefficient 95% CIs are from quantile regression at median. *ICU*, Intensive care unit; *CI*, confidence interval; *HLHS*, hypoplastic left heart syndrome.

∗ Significance level (*P* value): 0.01.

† 0.001.

‡ 0.1.

§ 0.05.

### Fontan Procedure Episode: Hospital Length of Stay and Factors Associated With Days in Intensive Care Unit

The median inpatient stay after the Fontan procedure was 13 days (IQR, 10-20; range, 1-273), and the median ICU stay after the Fontan procedure was 2 days (IQR, 1-3; range, 1-257). There were 23 (1.6%) in-hospital deaths, with nonsurvivors having a median spell length post-Fontan of 23 days (IQR, 3-57; range, 1-273) and median ICU stay of 22 days (IQR, 4-54; range, 1-257). One single extreme case (ICU stay of 257 days) required a heart transplant during the same protracted hospital stay.

There were 424 patients born after 2009 with PICANet ICU records included in this analysis, of whom 5 (1.2%) died after the Fontan procedure. None of the evaluated factors showed a significant association with ICU stay in univariable or multivariable regression.

### Six Months Post-Fontan: Inpatient Hospital Days and Factors Associated With Fewer Days at Home

In the first 6 months after the Fontan procedure, there were no additional inpatient episodes (spells) in 62.9% of patients, 1 in 22.7%, 2 in 8.4%, and 3 or more in 7% of patients. For those with at least 1 additional inpatient event, the median inpatient stay was 2 days (IQR, 1-6; range 1-114). There were 30 (2.1%) deaths within 6 months of a Fontan procedure, 22 being in-hospital deaths.

In the adjusted model (n = 419 patients born after 2009 with complete HES follow-up or death within 6 months of the Fontan procedure), severity of illness marker at any time before the Fontan operation (coefficient −9.1, CI, –13.2 to −5.3], *P* < .001) and more exposure to CPB during the first year of life (coefficient −1.3, CI, –2.2 to −0.5], *P* = .002) were associated with fewer days at home within 6 months of the Fontan procedure.

## Discussion

**Figure 4:**
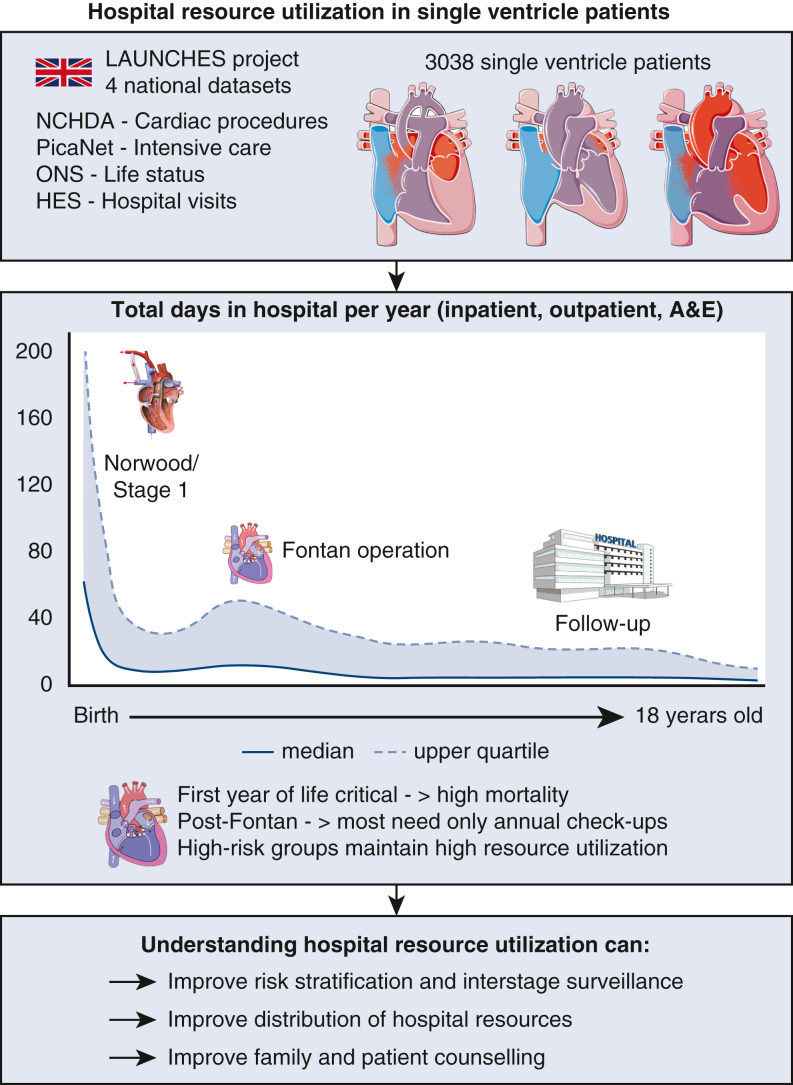
Understanding single ventricle resource use and at-risk populations can improve decision making, patient care, and counseling. *LAUCHES*, The Linking AUdit and National datasets in Congenital HEart Services; *NCHDA*, National Congenital Heart Disease Audit; *PICANet*, pediatric intensive care audit network; *ONS*, Office for National Statistics; *HES*, hospital episode statistics.

This analysis of hospital resource use and associated factors in patients with f-SV offers a comprehensive overview of hospital days from birth to 18 years of age (Figure 4), adding to previous work on f-SV survival and associated risk factors.^5^ The main finding is that after a critical first month of life when infants spend most of their time as inpatients, time spent in hospital gradually decreases. It is as low as 2 days/year (median) for an 18-year-old, transitioning to mostly outpatient-based care in adolescence. This shows a good clinical course for most Fontan procedure survivors, which is important for counseling.

Equally important but less optimistic, there are a group of patients with high first year mortality or requiring continued high levels of hospitalization and outpatient visits throughout their childhood and adolescence. Several risk factors have been identified and should be the focus of future research in these subgroups at risk. Given the significant overlap between these factors associated with hospital resource use in survivors and those previously reported to be linked to mortality,^5^ identifying measures to reduce their impact could result in both lower mortality and lower morbidity and hospital costs.

### The Hospital Journey of Patients With Functionally Single Ventricle From Birth to 18 Years Old

Management of f-SV is resource intensive in both hospital stay and financial costs,^7^, ^8^, ^9^, ^10^, ^11^^,^^15^ especially in Norwood nonsurvivor cases.^19^ Previous research has offered useful information on how hospital days are distributed throughout childhood for patients with f-SV, but not without limitations. The Philadelphia fetus-to-Fontan study reported a median of 21.5 days spent in hospital from birth to stage 1 and a median of 2 days for the interstage period;^8^ however, it lacks data beyond the Fontan and is limited to single-center follow-up. The Australia/New Zealand Fontan registry showed on average less than 40 in-hospital days spent during the first year of life and less than 10 days/year up to 18 years of age, but with limited details on how these means can be interpreted in the presence of right skewed data.^20^ Additionally, both studies focus on survivors of the Fontan procedure and do not differentiate well between inpatient and outpatient care.

The current study provides a highly detailed overview of hospital resource use in all f-SV patients with at least 1 cardiac procedure, from a large geographical area, from birth to 18 years of age, regardless of survival status up to or after the Fontan procedure, and with a differentiation between hospital visit type and cause. We found that time spent in hospital is concentrated during the first 2 months of life, when the highest mortality also occurs. Recent results have demonstrated improved interstage survival with continuous hospitalization,^30^ and it could be argued that a trade-off between fewer deaths and more hospital days could be acceptable.

Data on care needs beyond the first year of life are limited and difficult to generalize, because they focus on those who required hospitalizations^7^^,^^10^^,^^18^ and Fontan survivors,^20^ or provide an overall inpatient use up to 10 years of life but without other details.^21^ We found that after the age of 6 years, at least half the patients only require annual follow-up outpatient visits, with median inpatient days per year as low as 0, a lower burden than what was previously reported in older adults.^4^^,^^7^ This is reassuring, because hospitalizations for noncardiac causes in f-SV come with high mortality and healthcare costs.^9^^,^^18^

There remains considerable right skewness in terms of resource use needs, pointing to a subset of individuals with worse outcomes, driving the high mean values like those previously reported.^20^^,^^21^ The current analysis of factors associated with hospital resource use during the initial f-SV palliation builds on previous studies that identified right ventricle anatomy, low weight, and critical preoperative status as predictors of various measures of hospital resource use, although with smaller sample sizes of 202 to 409 patients,^8^, ^16^ as well as a comprehensive analysis of mortality-associated risk factors using the LAUNCHES dataset.^5^ The analysis of risk factors accounted for possible center variation, but in a centralized system such as that present in the NHS, the impact of individual center practice on outcomes is low, as recently shown in a study by our group.^5^ With further independent replication, comparisons with other forms of CHD and measures of discrimination and calibration, these factors could identify subgroups with differing requirements for monitoring and follow-up. These in turn could be targets for specific research with the aim of implementing quality improvements to address the difference in outcomes demonstrated here.

### Informing Patients and Families

In the era of antenatal diagnosis of CHD, discussions on expected outcomes are crucial and require detailed information. Parents' concerns go beyond survival into how their daily life and that of their children will be affected, among other things, by time spent in the hospital.^22^, ^27^

A diagnosis of f-SV entails a well-described early risk of death, but information on other aspects of later care is scarce. The current data aim to provide a tool to inform the parents and later the patients themselves. Expectations can be managed based on hard evidence resulting from a national registry, with figures by age intervals, taking into consideration factors we identified to be associated with fewer days at home.

### Study Limitations

This is an analysis of linked national registries, and thus it is limited by data availability but has the advantage of external validation. Analyses were limited to subsets with complete data, leading to exclusions of some patients from certain analyses, preferred to missing data imputation to avoid misclassification. Patient selection was procedure based, excluding cases of neonatal compassionate care or deaths before any intervention. By allocating 0 days at home on deaths in the days-at-home analysis, more weight is given to these cases in the quantile regression, although this is mitigated by the fact that they died predominately in-hospital. When comparing hospital resource use at different age intervals, survivor bias is inherent, with earlier deaths reflecting the most severe cases. Patients living in Wales might have underestimation of late hospital resource use, because noncardiac local follow-up might not be accurately captured, although these are a small percentage of the total group. There were some ambiguous care episodes (spells) not classifiable as cardiac or noncardiac, where diagnoses were not noted, and instead the general term “follow-up” was used, reported separately. The analysis on factors associated with health resource use was exploratory, and as such causality should not be inferred from it.

## Conclusions

This study offers a detailed description of length and type of hospital stay from birth to 18 years and patient-level factors associated with hospital resource use to aid in clinical decision making and counseling. Most of the hospital stay and resource use are concentrated in the first 2 months of life and decrease substantially over time, transitioning from a predominately inpatient care to mostly outpatient visits. There are those with poor outcomes, including nonsurvivors, and those with high resource use persisting until the age of 18 years, where identifying modifiable factors could improve quality of care and help narrow the gap in outcomes.

### Conflict of Interest Statement

D.A.L. has received support from Roach Diagnostics and 10.13039/100004374Medtronic Ltd for research unrelated to that presented here. All other authors reported no conflicts of interest.

The *Journal* policy requires editors and reviewers to disclose conflicts of interest and to decline handling or reviewing manuscripts for which they may have a conflict of interest. The editors and reviewers of this article have no conflicts of interest.

## Figures and Tables

**Table E1:** Diagnostic codes indicative of HLHS related malformations

03.01.05	Left isomerism (polysplenia)
03.01.04	Right isomerism (asplenia)
01.03.09	Atrioventricular or ventriculo-arterial connections abnormal
01.01.14	Double inlet atrioventricular connection (double inlet ventricle)
01.04.03	Double inlet right ventricle
01.04.04	Double inlet left ventricle
06.01.01	Tricuspid atresia
01.05.01	Discordant ventriculo-arterial connections, transposition of great arteries (TGA)
01.01.02	TGA (concordant atrioventricular and discordant ventriculo-arterial connections) and intact ventricular septum
01.01.03	Congenitally corrected transposition of great arteries (discordant atrioventricular and ventriculo-arterial connections)
01.01.04	Double outlet right ventricle
01.01.17	Double outlet right ventricle: Fallot type (subaortic or doubly committed ventricular septal defect and pulmonary stenosis)
01.01.40	Double outlet right ventricle: subaortic or doubly committed ventricular septal defect without pulmonary stenosis (ventricular septal defect type)
01.01.18	Double outlet right ventricle: transposition type (subpulmonary ventricular septal defect)
01.01.19	Double outlet right ventricle: with noncommitted ventricular septal defect
01.01.24	Double outlet right ventricle: with intact ventricular septum
01.05.03	Double outlet left ventricle
09.01.01	Common arterial trunk (truncus arteriosus)
09.05.11	Pulmonary atresia
09.05.12	Pulmonary atresia: imperforate valve
01.01.07	Pulmonary atresia + intact ventricular septum
01.01.06	Pulmonary atresia + ventricular septal defect (including Fallot type)
01.01.25	Pulmonary atresia + ventricular septal defect + systemic-to-pulmonary collateral artery(ies) (major aortopulmonary collateral arteries)
06.02.09	Straddling mitral valve
06.06.00	Atrioventricular septal defect (AVSD)
06.06.01	Atrioventricular septal defect (ASD): isolated atrial component (primum ASD) (partial AVSD)
06.06.08	Atrioventricular septal defect: isolated ventricular component
06.06.10	Atrioventricular septal defect: atrial and (restrictive) ventricular components + separate atrioventricular valve orifices (‘intermediate')
06.06.09	Atrioventricular septal defect: atrial and ventricular components with common atrioventricular orifice (complete)
01.01.20	Atrioventricular septal defect and tetralogy of Fallot
06.07.26	Atrioventricular septal defect with ventricular imbalance
06.05.01	Atrioventricular septal defect atrioventricular valvar abnormality
06.05.06	Atrioventricular septal defect atrioventricular valvar regurgitation
07.08.41	Ventricular imbalance: dominant left ventricle + hypoplastic right ventricle
07.02.00	Right ventricular hypoplasia

**Table E2:** Diagnosis codes indicative of functional univentricular heart

01.01.14	Double inlet atrioventricular connection (double inlet ventricle)
01.01.22	Functionally univentricular heart
01.01.24	Double outlet right ventricle with intact ventricular septum
01.04.03	Double inlet right ventricle
01.04.04	Double inlet left ventricle
02.03.05	Solitary ventricle of indeterminate morphology
06.01.01	Tricuspid atresia
06.07.26	Atrioventricular septal defect with ventricular imbalance
07.08.41	Ventricular imbalance: dominant left ventricle + hypoplastic right ventricle
07.08.42	Ventricular imbalance: dominant right ventricle and HLV

*HLV,* Hypoplastic left ventricle.

**Table E3:** Diagnosis codes indicative of pulmonary atresia, common arterial trunk or other variants not classifiable as functional univentricular heart

01.01.07	Pulmonary atresia + intact ventricular septum
01.01.25	Pulmonary atresia + ventricular septal defect + systemic-to-pulmonary collateral artery(ies) (major aortopulmonary collateral arteries)
01.01.20	Atrioventricular septal defect and tetralogy of Fallot
09.05.25	Absent pulmonary valve syndrome
09.01.01	Common arterial trunk (truncus arteriosus)

**Table E4:** Number of linked records in each dataset by financial year

Financial year	NCHDA	PICANet	A&E	APC	Outpatient
2000	200	0	0	555	0
2001	258	2	0	877	0
2002	271	94	0	1137	0
2003	379	335	0	1526	2499
2004	446	422	0	1838	3348
2005	478	513	0	2195	4243
2006	592	591	0	2670	5614
2007	623	629	618	3185	7484
2008	657	624	656	2957	8838
2009	724	717	943	3599	10,878
2010	769	738	1045	3833	13,361
2011	798	767	1116	3974	14,641
2012	774	687	1233	3863	17,490
2013	887	812	1431	4144	19,391
2014	993	787	1707	4488	24,882
2015	1256	794	1901	4465	27,798
2016	1136	629	1870	4188	26,848
2017	0	0	1768	3203	24,825
Total	11,241	9141	14,288	52,697	212,140

*NCHDA*, National Congenital Heart Disease Audit; *PICANet*, pediatric intensive care audit network; *A&E*, accidents and emergency department; *APC*, admitted patient care or hospitalization.

**Table E5:** Number of days/month spent in hospital (inpatient, outpatient and A&E not admitted) by age intervals during the first year of life (numerical data for manuscript Figure 2, *A*)

Age interval (mo)	Days/mo						Patient No.		
	Mean			Median [IQR] (min-max range)					
	Overall	Cardiac	Noncardiac/ambiguous	Overall	Cardiac	Noncardiac/ambiguous	Overall	Death (%)	Censoring (%)
(0, 1)	21.3	20.6	0.7	25 [14, 30] (0-30)	24 [13, 30] (0-30)	0 [0, 0] (0-30)	1828	145 (7.9)	0 (0)
(1, 2)	12.2	11.2	1	8 [2, 24] (0-30)	6 [1, 23] (0-30)	0 [0, 1] (0-30)	1705	73 (4.3)	0 (0)
(2, 3)	9	7.9	1.1	4 [1, 14] (0-30)	2 [1, 12] (0-30)	0 [0, 1] (0-30)	1647	45 (2.7)	0 (0)
(3, 4)	8.5	7.5	1.1	3 [1, 12] (0-30)	2 [1, 11] (0-30)	0 [0, 1] (0-30)	1615	42 (2.6)	0 (0)
(4, 5)	7.5	6.5	1	3 [1, 10] (0-30)	1 [0, 9] (0-30)	0 [0, 1] (0-28)	1584	27 (1.7)	0 (0)
(5, 6)	6.8	5.8	1	3 [1, 9] (0-30)	1 [0, 7] (0-30)	0 [0, 1] (0-30)	1568	21 (1.3)	0 (0)
(6, 7)	5.6	4.7	1	2 [1, 6] (0-30)	1 [0, 4] (0-30)	0 [0, 1] (0-30)	1558	14 (0.9)	0 (0)
(7, 8)	4.8	3.8	1	2 [0, 5] (0-30)	1 [0, 2] (0-30)	0 [0, 1] (0-30)	1567	14 (0.9)	0 (0)
(8, 9)	4	3.1	1	1 [1, 3] (0-30)	1 [0, 1] (0-30)	0 [0, 1] (0-30)	1568	12 (0.8)	0 (0)
(9, 10)	3.4	2.4	0.9	1 [0, 3] (0-30)	0 [0, 1] (0-30)	0 [0, 1] (0-30)	1562	8 (0.5)	0 (0)
(10, 11)	3	2.1	0.9	1 [0, 3] (0-30)	0 [0, 1] (0-30)	0 [0, 1] (0-30)	1572	18 (1.1)	0 (0)
(11, 12)	2.4	1.6	0.8	1 [0, 2] (0-30)	0 [0, 1] (0-30)	0 [0, 1] (0-24)	1568	4 (0.3)	0 (0)

Patients were included in each age interval if had HES data (inpatient, outpatient and A&E) and were alive or not censored in HES in the lower age limit. Exclude patients on the age intervals if have inpatient spells without a discharge date. Patients not linked to HES were excluded from the analysis. *IQR*, Interquartile range.

**Table E6:** Number of days/month spent in hospital (inpatient) by age intervals during the first year of life (numerical data for manuscript Figure 2, *B*)

Age interval (mo)	Days/mo						Patient No.		
	Mean			Median [IQR] (min-max range)					
	Overall	Cardiac	Noncardiac/ambiguous	Overall	Cardiac	Noncardiac/ambiguous	Overall	Death (%)	Censoring (%)
(0, 1)	19.9	19.2	0.6	22 [11, 30] (0-30)	22 [10, 30] (0-30)	0 [0, 0] (0-30)	2827	202 (7.1)	0 (0)
(1, 2)	10.1	9.6	0.5	4 [0, 19] (0-30)	3 [0, 18] (0-30)	0 [0, 0] (0-30)	2639	97 (3.7)	0 (0)
(2, 3)	6.8	6.3	0.5	1 [0, 10] (0-30)	0 [0, 8] (0-30)	0 [0, 0] (0-30)	2542	61 (2.4)	0 (0)
(3, 4)	6.3	5.7	0.5	1 [0, 8] (0-30)	0 [0, 7] (0-30)	0 [0, 0] (0-30)	2481	53 (2.1)	0 (0)
(4, 5)	5.5	5	0.5	0 [0, 6] (0-30)	0 [0, 6] (0-30)	0 [0, 0] (0-27)	2428	36 (1.5)	0 (0)
(5, 6)	5	4.5	0.5	0 [0, 6] (0-30)	0 [0, 4] (0-30)	0 [0, 0] (0-30)	2392	32 (1.3)	0 (0)
(6, 7)	4.1	3.6	0.5	0 [0, 3] (0-30)	0 [0, 2] (0-30)	0 [0, 0] (0-30)	2360	21 (0.9)	0 (0)
(7, 8)	3.4	2.9	0.5	0 [0, 2] (0-30)	0 [0, 1] (0-30)	0 [0, 0] (0-30)	2339	17 (0.7)	0 (0)
(8, 9)	2.7	2.3	0.4	0 [0, 1] (0-30)	0 [0, 0] (0-30)	0 [0, 0] (0-30)	2322	16 (0.7)	0 (0)
(9, 10)	2.1	1.8	0.4	0 [0, 1] (0-30)	0 [0, 0] (0-30)	0 [0, 0] (0-30)	2306	15 (0.7)	0 (0)
(10, 11)	2	1.5	0.4	0 [0, 1] (0-30)	0 [0, 0] (0-30)	0 [0, 0] (0-30)	2291	22 (1)	0 (0)
(11, 12)	1.6	1.3	0.3	0 [0, 0] (0-30)	0 [0, 0] (0-30)	0 [0, 0] (0-28)	2269	7 (0.3)	0 (0)

Patients were included in each age interval if alive or not censored in HES in the lower age limit. Exclude patients on the age intervals if have inpatient spells without a discharge date. Patients not linked to HES were excluded from the analysis. *IQR*, Interquartile range.

**Table E7:** Number of days/month spent in intensive care unit by age intervals during the first year of life (numerical data for manuscript Figure 2, *C*)

Age interval (mo)	Days/mo		Patient No.		
	Mean	Median [IQR] (min-max range)	Overall	Death (%)	Censoring (%)
(0, 1)	8.1	6 [1, 12] (0-30)	2726	240 (8.8)	9 (0.3)
(1, 2)	2.6	0 [0, 1] (0-30)	2477	99 (4)	4 (0.2)
(2, 3)	1.8	0 [0, 0] (0-30)	2374	61 (2.6)	6 (0.3)
(3, 4)	1.7	0 [0, 0] (0-30)	2307	53 (2.3)	12 (0.5)
(4, 5)	1.4	0 [0, 0] (0-30)	2242	32 (1.4)	4 (0.2)
(5, 6)	1.2	0 [0, 0] (0-30)	2206	31 (1.4)	12 (0.5)
(6, 7)	0.9	0 [0, 0] (0-30)	2163	22 (1)	17 (0.8)
(7, 8)	0.7	0 [0, 0] (0-30)	2124	15 (0.7)	11 (0.5)
(8, 9)	0.6	0 [0, 0] (0-30)	2098	16 (0.8)	11 (0.5)
(9, 10)	0.4	0 [0, 0] (0-30)	2071	14 (0.7)	15 (0.7)
(10, 11)	0.4	0 [0, 0] (0-30)	2042	22 (1.1)	12 (0.6)
(11, 12)	0.3	0 [0, 0] (0-30)	2008	6 (0.3)	4 (0.2)

Patients were included in each age interval if had PICANet data (born since 2002) and were alive or not censored in PICANet in the lower age limit. Patients not linked to PICANet were excluded from the analysis. *IQR*, Interquartile range.

**Table E8:** Number of days/month spent in hospital (outpatient) by age intervals during the first year of life (numerical data for manuscript Figure 2, *B*)

Age interval (mo)	Days/mo						Patient No.		
	Mean			Median [IQR] (min-max range)					
	Overall	Cardiac	Noncardiac/ambiguous	Overall	Cardiac	Noncardiac/ambiguous	Overall	Death (%)	Censoring (%)
(0, 1)	0.3	0.2	0.1	0 [0, 0] (0-10)	0 [0, 0] (0-5)	0 [0, 0] (0-8)	2514	195 (7.8)	0 (0)
(1, 2)	1	0.6	0.4	1 [0, 2] (0-10)	0 [0, 1] (0-9)	0 [0, 0] (0-9)	2324	94 (4)	0 (0)
(2, 3)	1	0.6	0.5	1 [0, 1] (0-9)	0 [0, 1] (0-7)	0 [0, 1] (0-8)	2242	60 (2.7)	0 (0)
(3, 4)	1	0.6	0.5	1 [0, 1] (0-10)	0 [0, 1] (0-8)	0 [0, 1] (0-7)	2191	51 (2.3)	0 (0)
(4, 5)	1	0.5	0.4	1 [0, 1] (0-10)	0 [0, 1] (0-7)	0 [0, 1] (0-8)	2148	32 (1.5)	0 (0)
(5, 6)	1	0.5	0.5	1 [0, 1] (0-12)	0 [0, 1] (0-9)	0 [0, 1] (0-10)	2124	32 (1.5)	0 (0)
(6, 7)	0.9	0.5	0.4	1 [0, 1] (0-9)	0 [0, 1] (0-7)	0 [0, 1] (0-9)	2101	21 (1)	0 (0)
(7, 8)	0.9	0.4	0.4	1 [0, 1] (0-10)	0 [0, 1] (0-10)	0 [0, 1] (0-8)	2091	16 (0.8)	0 (0)
(8, 9)	0.9	0.4	0.4	1 [0, 1] (0-12)	0 [0, 1] (0-12)	0 [0, 1] (0-11)	2082	16 (0.8)	0 (0)
(9, 10)	0.8	0.4	0.4	0 [0, 1] (0-9)	0 [0, 1] (0-8)	0 [0, 1] (0-9)	2072	14 (0.7)	0 (0)
(10, 11)	0.8	0.4	0.4	0 [0, 1] (0-9)	0 [0, 1] (0-9)	0 [0, 1] (0-9)	2066	22 (1.1)	0 (0)
(11, 12)	0.7	0.3	0.4	0 [0, 1] (0-11)	0 [0, 1] (0-11)	0 [0, 1] (0-9)	2055	6 (0.3)	0 (0)

Patients were included in each age interval if had outpatient data and were alive or not censored in HES in the lower age limit. Patients not linked to HES were excluded from the analysis. *IQR*, Interquartile range.

**Table E9:** Number of days/year spent in hospital (inpatient, outpatient and A&E not admitted) by age intervals between 0 and 18 years (numerical data for manuscript Figure 4, *A*)

Age interval (y)	Days/y						Patient No.		
	Mean			Median [IQR] (min-max range)					
	Overall	Cardiac	Noncardiac/ambiguous	Overall	Cardiac	Noncardiac/ambiguous	Overall	Death (%)	Censoring (%)
(0, 1)	79.4	69.6	9.8	60 [37, 102] (0-365)	50 [30, 86] (0-365)	6 [1, 13] (0-255)	1826	414 (22.7)	0 (0)
(1, 2)	17.5	9.8	7.6	9 [5, 19] (0-365)	3 [2, 8] (0-365)	4 [2, 9] (0-114)	1580	40 (2.5)	100 (6.3)
(2, 3)	11.9	6.8	5.2	6 [3, 14] (0-356)	2 [1, 5] (0-356)	3 [1, 6] (0-73)	1570	22 (1.4)	130 (8.3)
(3, 4)	14.3	9.2	5.1	7 [3, 18] (0-365)	3 [2, 10] (0-365)	2 [1, 6] (0-67)	1540	15 (1)	131 (8.5)
(4, 5)	16	10.4	5.5	9 [4, 22] (0-360)	3 [2, 14] (0-360)	3 [1, 7] (0-79)	1504	12 (0.8)	153 (10.2)
(5, 6)	14	8.6	5.5	7 [3, 19] (0-302)	2 [1, 8] (0-302)	3 [1, 7] (0-66)	1432	9 (0.6)	124 (8.7)
(6, 7)	11.3	6.4	4.9	6 [2, 15] (0-365)	2 [1, 4] (0-365)	2 [0, 6] (0-94)	1386	6 (0.4)	144 (10.4)
(7, 8)	9.3	4.8	4.6	4 [2, 13] (0-115)	2 [1, 3] (0-115)	2 [0, 6] (0-62)	1342	6 (0.4)	139 (10.4)
(8, 9)	8.5	4	4.4	4 [2, 11] (0-120)	1 [1, 3] (0-109)	2 [0, 5] (0-76)	1197	4 (0.3)	151 (12.6)
(9, 10)	8.5	4.1	4.4	4 [2, 11] (0-131)	1 [1, 3] (0-129)	2 [0, 6] (0-66)	1042	8 (0.8)	121 (11.6)
(10, 11)	8.4	3.9	4.5	4 [1, 11] (0-135)	1 [1, 3] (0-70)	2 [0, 6] (0-115)	913	6 (0.7)	144 (15.8)
(11, 12)	7.8	3.7	4.1	4 [1, 10] (0-239)	1 [1, 3] (0-239)	2 [0, 5] (0-50)	763	2 (0.3)	141 (18.5)
(12, 13)	7.9	3.9	4	4 [1, 9] (0-123)	1 [1, 3] (0-111)	2 [0, 5] (0-62)	620	2 (0.3)	127 (20.5)
(13, 14)	7.6	3.8	3.8	3 [1, 9] (0-218)	1 [1, 2] (0-210)	1 [0, 4] (0-54)	491	0 (0)	110 (22.4)
(14, 15)	7.4	3.5	3.9	3 [1, 9] (0-83)	1 [0, 3] (0-74)	1 [0, 5] (0-46)	381	0 (0)	100 (26.2)
(15, 16)	6.9	3	3.9	3 [1, 8] (0-59)	1 [1, 3] (0-33)	1 [0, 4] (0-54)	281	0 (0)	93 (33.1)
(16, 17)	5.2	2.2	3	2 [1, 6] (0-35)	1 [1, 2] (0-24)	1 [0, 4] (0-31)	188	0 (0)	86 (45.7)
(17, 18)	4.1	1.5	2.6	2 [0, 5] (0-44)	1 [0, 2] (0-38)	0 [0, 3] (0-43)	102	0 (0)	102 (100)

Patients were included in each age interval if had HES data (inpatient, outpatient, and A&E) and were alive or not censored in HES in the lower age limit. Exclude patients on the age intervals if have inpatient spells without a discharge date. Patients not linked to HES were excluded from the analysis. *IQR*, Interquartile range.

**Table E10:** Number of days/year spent in hospital (inpatient) by age intervals between 0 and 18 years (numerical data for manuscript Figure 4, *B*)

Age interval (y)	Days/y						Patient No.		
	Mean			Median [IQR] (min-max range)					
	Overall	Cardiac	Noncardiac/ambiguous	Overall	Cardiac	Noncardiac/ambiguous	Overall	Death (%)	Censoring (%)
(0, 1)	62.6	57.6	5	43 [25, 77] (0-365)	39 [22, 69] (0-365)	1 [0, 5] (0-254)	2819	576 (20.4)	0 (0)
(1, 2)	10	7.3	2.7	2 [0, 9] (0-365)	0 [0, 6] (0-365)	1 [0, 2] (0-102)	2262	57 (2.5)	100 (4.4)
(2, 3)	5.8	4.3	1.5	1 [0, 4] (0-365)	0 [0, 2] (0-365)	0 [0, 1] (0-62)	2104	28 (1.3)	130 (6.2)
(3, 4)	7.7	6.3	1.3	2 [0, 9] (0-365)	1 [0, 6] (0-365)	0 [0, 1] (0-70)	1946	18 (0.9)	131 (6.7)
(4, 5)	8.2	7	1.2	2 [0, 11] (0-360)	1 [0, 10] (0-360)	0 [0, 1] (0-50)	1798	17 (0.9)	153 (8.5)
(5, 6)	6.2	5.2	0.9	1 [0, 5] (0-302)	0 [0, 3] (0-302)	0 [0, 1] (0-45)	1628	9 (0.6)	124 (7.6)
(6, 7)	4.2	3.3	0.8	0 [0, 2] (0-365)	0 [0, 1] (0-365)	0 [0, 1] (0-75)	1493	7 (0.5)	144 (9.6)
(7, 8)	2.7	2.1	0.6	0 [0, 1] (0-115)	0 [0, 0] (0-114)	0 [0, 0] (0-57)	1342	6 (0.4)	139 (10.4)
(8, 9)	2.1	1.4	0.6	0 [0, 1] (0-103)	0 [0, 0] (0-100)	0 [0, 0] (0-51)	1197	4 (0.3)	151 (12.6)
(9, 10)	2.1	1.5	0.6	0 [0, 1] (0-125)	0 [0, 0] (0-125)	0 [0, 0] (0-52)	1042	8 (0.8)	121 (11.6)
(10, 11)	2	1.2	0.7	0 [0, 1] (0-119)	0 [0, 0] (0-63)	0 [0, 0] (0-101)	913	6 (0.7)	144 (15.8)
(11, 12)	1.5	0.9	0.6	0 [0, 1] (0-239)	0 [0, 0] (0-238)	0 [0, 0] (0-38)	763	2 (0.3)	141 (18.5)
(12, 13)	1.9	1.4	0.5	0 [0, 1] (0-109)	0 [0, 0] (0-107)	0 [0, 0] (0-22)	620	2 (0.3)	127 (20.5)
(13, 14)	1.6	1	0.6	0 [0, 0] (0-207)	0 [0, 0] (0-202)	0 [0, 0] (0-47)	491	0 (0)	110 (22.4)
(14, 15)	1.3	0.7	0.5	0 [0, 1] (0-67)	0 [0, 0] (0-60)	0 [0, 0] (0-20)	381	0 (0)	100 (26.2)
(15, 16)	1.5	0.8	0.7	0 [0, 1] (0-48)	0 [0, 0] (0-27)	0 [0, 0] (0-47)	281	0 (0)	93 (33.1)
(16, 17)	0.8	0.3	0.5	0 [0, 0] (0-32)	0 [0, 0] (0-11)	0 [0, 0] (0-28)	188	0 (0)	86 (45.7)
(17, 18)	0.8	0.6	0.2	0 [0, 0] (0-36)	0 [0, 0] (0-35)	0 [0, 0] (0-9)	102	0 (0)	102 (100)

Patients were included in each age interval if alive or not censored in HES in the lower age limit. Exclude patients on the age intervals if have inpatient spells without a discharge date. Patients not linked to HES were excluded from the analysis. *IQR*, Interquartile range.

**Table E11:** Number of days/year spent in intensive care unit by age intervals between 0 and 15 years∗ (numerical data for manuscript Figure 4, *C*)

Age interval (y)	Days/y		Patient No.		
	Mean	Median [IQR] (min-max range)	Overall	Death (%)	Censoring (%)
(0, 1)	8.1	10 [4, 20] (0-365)	2726	611 (22.4)	117 (4.3)
(1, 2)	2.6	0 [0, 1] (0-141)	1998	51 (2.6)	134 (6.7)
(2, 3)	1.8	0 [0, 0] (0-89)	1813	18 (1)	146 (8.1)
(3, 4)	1.7	0 [0, 1] (0-225)	1649	17 (1)	157 (9.5)
(4, 5)	1.4	0 [0, 1] (0-158)	1475	13 (0.9)	137 (9.3)
(5, 6)	1.2	0 [0, 0] (0-36)	1325	8 (0.6)	153 (11.5)
(6, 7)	0.9	0 [0, 0] (0-39)	1164	6 (0.5)	145 (12.5)
(7, 8)	0.7	0 [0, 0] (0-71)	1013	5 (0.5)	152 (15)
(8, 9)	0.6	0 [0, 0] (0-52)	856	2 (0.2)	123 (14.4)
(9, 10)	0.4	0 [0, 0] (0-90)	731	3 (0.4)	146 (20)
(10, 11)	0.4	0 [0, 0] (0-43)	582	5 (0.9)	144 (24.7)
(11, 12)	0.3	0 [0, 0] (0-3)	433	2 (0.5)	125 (28.9)
(12, 13)	8.1	0 [0, 0] (0-91)	306	1 (0.3)	113 (36.9)
(13, 14)	2.6	0 [0, 0] (0-17)	192	0 (0)	100 (52.1)
(14, 15)	1.8	0 [0, 0] (0-7)	92	0 (0)	92 (100)

*IQR*, Interquartile range.

∗ No PICANet data on age more than 15 years. Patients were included in each age interval if had PICANet data (born since 2002) and were alive or not censored in PICANet in the lower age limit. Patients not linked to PICANet were excluded from the analysis.

**Table E12:** Number of days/year spent in hospital (outpatient) by age intervals between 0 and 18 years (numerical data for manuscript Figure 4, *D*)

Age interval (y)	Days/y						Patient No.		
	Mean			Median [IQR] (min-max range)					
	Overall	Cardiac	Noncardiac/ambiguous	Overall	Cardiac	Noncardiac/ambiguous	Overall	Death (%)	Censoring (%)
(0, 1)	8.8	4.7	4.1	8 [3, 12] (0-75)	4 [1, 7] (0-55)	2 [0, 6] (0-70)	2514	557 (22.2)	0 (0)
(1, 2)	6.6	2.8	3.8	5 [3, 8] (0-86)	2 [1, 4] (0-49)	2 [0, 5] (0-77)	2058	52 (2.5)	100 (4.9)
(2, 3)	5.2	2.2	2.9	4 [2, 6] (0-63)	2 [1, 3] (0-38)	2 [0, 4] (0-57)	1999	26 (1.3)	130 (6.5)
(3, 4)	5.5	2.5	3	4 [2, 6] (0-67)	2 [1, 3] (0-66)	1 [0, 4] (0-52)	1951	18 (0.9)	132 (6.8)
(4, 5)	6.5	2.9	3.6	4 [2, 8] (0-80)	2 [1, 3] (0-80)	2 [0, 4] (0-60)	1801	17 (0.9)	153 (8.5)
(5, 6)	7	3	4	4 [2, 9] (0-55)	2 [1, 3] (0-50)	2 [0, 5] (0-51)	1631	9 (0.6)	125 (7.7)
(6, 7)	6.4	2.8	3.6	4 [2, 8] (0-56)	2 [1, 3] (0-51)	1 [0, 4] (0-40)	1497	7 (0.5)	147 (9.8)
(7, 8)	6.3	2.7	3.6	3 [1, 8] (0-54)	1 [1, 2] (0-41)	1 [0, 4] (0-52)	1343	6 (0.4)	139 (10.3)
(8, 9)	6	2.6	3.5	3 [1, 8] (0-66)	1 [1, 2] (0-38)	1 [0, 4] (0-65)	1198	4 (0.3)	151 (12.6)
(9, 10)	6.1	2.6	3.5	3 [1, 8] (0-49)	1 [1, 2] (0-44)	1 [0, 4] (0-47)	1043	8 (0.8)	121 (11.6)
(10, 11)	6.1	2.7	3.5	3 [1, 8] (0-59)	1 [1, 2] (0-58)	1 [0, 4] (0-46)	914	6 (0.7)	145 (15.9)
(11, 12)	6	2.8	3.3	3 [1, 7] (0-67)	1 [1, 2] (0-67)	1 [0, 4] (0-41)	763	2 (0.3)	141 (18.5)
(12, 13)	5.7	2.5	3.2	3 [1, 7] (0-70)	1 [1, 2] (0-69)	1 [0, 4] (0-62)	620	2 (0.3)	127 (20.5)
(13, 14)	5.7	2.7	3	3 [1, 7] (0-81)	1 [1, 2] (0-76)	1 [0, 3] (0-44)	491	0 (0)	110 (22.4)
(14, 15)	5.8	2.7	3	3 [1, 7] (0-52)	1 [0, 2] (0-51)	1 [0, 3] (0-35)	381	0 (0)	100 (26.2)
(15,16)	5.1	2.2	2.9	3 [1, 6] (0-32)	1 [1, 2] (0-25)	1 [0, 3] (0-30)	281	0 (0)	93 (33.1)
(16, 17)	4.1	1.8	2.3	2 [1, 5] (0-27)	1 [0, 2] (0-20)	0 [0, 2] (0-25)	188	0 (0)	86 (45.7)
(17, 18)	3.1	0.9	2.3	1 [0, 4] (0-44)	1 [0, 1] (0-5)	0 [0, 2] (0-43)	102	0 (0)	102 (100)

Patients were included in each age interval if had outpatient data and were alive or not censored in HES in the lower age limit. Patients not linked to HES were excluded from the analysis. *IQR*, Interquartile range.

## Appendix E1 Supplementary Methods

### Data Management

Each dataset has a different start date and end date (Table E4). This results in each analysis performed for periods when all required datasets overlapped, which is mentioned at each respective step. In addition, when describing hospital resource use, left truncation occurs where data on days spent in hospital/outpatient/A&E were available only from a given age onward. In the descriptive analysis, patients were included in age intervals where there were data available for the respective interval. Some patients present in the lower age limit of age intervals but die or are censored before the upper age limit and did not have complete hospital data use (right censoring). These age intervals were included in the descriptive analysis.

Before 2009, preoperative risk factors were less completely collected in the NCHDA dataset. For this reason, the univariable and multivariable analysis of factors associated with hospital resource use only included data from 2009 onwards, and their descriptive analysis also reflects this.

In addition, PICANet uses an internal censoring mechanism, where entries of patients aged more than 18 years can no longer be identified, so those born before 2002 were not linked.

### Patient Care Spells Definition

All clinical data were organized into patient care episodes or “spells,” containing patient events separated by no more than 24 hours.^23^ A spell encompasses a single overall health care interaction that can range from a single outpatient appointment or a prolonged inpatient stay. A spell was considered cardiac if it contains an NCHDA procedure or at least 1 cardiac diagnosis or healthcare resource group code for an HES inpatient stay, outpatient, or A&E visit.

### Patient Selection and Classification

The inclusion and exclusion steps are summarized in Figure 1. All patients undergoing a cardiac procedure from the NCHDA audit, born after April 2000 (to ensure procedural history completeness), were considered. To allow for accurate follow-up only those from England and Wales and treated within the National Health Service were included.

Cardiac diagnosis and procedure codes^24^ were used to identify patients with f-SV using a hierarchical series of steps: First, those undergoing biventricular repair were excluded;^2^ next, a stepwise algorithm was used to identify HLHS^6^ and non-HLHS subtypes^5^ (details in Appendix E1). Eight hierarchical diagnosis subgroups were created having at least 100 patients: HLHS, f-SV with any type of atrial isomerism, double inlet left ventricle, tricuspid atresia, unbalanced common atrioventricular canal defect, mitral atresia without HLHS, pulmonary atresia without other complex features but with a functionally SV circulation and a remaining “all other f-SV.”

### Data Collection and Management

The following data were extracted from the available datasets (details by year and data source in Table E4): *Data related to patients*: age and weight at first cardiac procedure and at Fontan operation, gender, anatomic diagnosis subtype,^2^, ^6^ and preoperative risk factors as used by the UK Partial Risk Adjustment in Surgery model for 30-day mortality (2009 and after).^25^ Based on weight-for-age Z scores,^E1^ those records with a Z score greater than 5 (abnormally high weight) were marked as missing (considered a data entry error), whereas records with Z score values less than −8 (abnormally low weight) were treated as missing only after being reviewed by the study team.

#### Data related to procedures

Year of procedure, type of stage 1 procedure,^2^ whether a stage 2 procedure is performed, whether a Fontan procedure is performed, number of off-pathway procedures performed,^2^, ^6^ total CPB time in the first year of life (as a surrogate for number and complexity of cumulated surgical procedures), Fontan operation crossclamp duration.

### Outcomes

#### Hospital resource use outcomes

1. Days in hospital: total (inpatient, outpatient, and A&E), inpatient, outpatient, and ICU days during the first year of life (1-month age intervals); 2. days in hospital: total (inpatient, outpatient and A&E), inpatient, outpatient and ICU days from 0 to 18 years (1-year age intervals); 3. Days at home during the first year of life; 4. inpatient spell length and ICU days post-Fontan procedure; 5. inpatient days and days at home in the first 6 months post-Fontan procedure. Spell length and ICU length of less than 1 day are counted as 1 day.

#### Vital status

Patient life status was provided at the point of hospital discharge by NCHDA for each cardiac procedure and at date of extract by the Office of National Statistics (national death registry data). Death recorded in either NCHDA or ONS was counted as a death, but if death was recorded in both, date of death from NCHDA was used (if there was disagreement). Any patients who were discharged alive in NCHDA and who have missing life status with ONS were deemed lost to follow-up and were censored at their last known live discharge age.

### Statistical Analyses

Frequencies are reported as numbers and proportions, and continuous variables as median with IQR and minimum-maximum range, as well as means in Table E10, Table E11, Table E12, Table E5, Table E6, Table E7, Table E8, Table E9. Any missing values affecting the denominator or leading to exclusions from analyses are detailed where relevant and were not imputed due to high level of data completeness.

### Hospital Resource Use (First Year of Life and 0-18 Years)

Median (IQR) days in hospital/ICU were counted within each age interval. Intervals of 1 month were used for the first year of life and 1-year age intervals were used for 2 to 18 years. The numbers of patients who died or were right-censored within each interval are reported (Table E10, Table E11, Table E12, Table E5, Table E6, Table E7, Table E8, Table E9).

### Factors Associated with Days at Home and ICU Time Analysis

To investigate factors associated with hospital resource use, 4 outcomes of interest were used: days at home and days in ICU during first year of life, postoperative ICU days during the Fontan spell of care, and days at home within 6 months post-Fontan. Patients who died before year 1/within 6 months of a Fontan procedure were assigned a value of zero days at home as the worst outcome.^26^, ^27^ Patients censored in HES/PICANet during the first year of life (or within 6 months of Fontan procedure) were excluded from the respective days at home/days in ICU analyses.

Quantile regression was carried out to explore factors associated with the median of each outcome of interest, as the data are highly skewed.^28^ Effects are presented as univariable and adjusted coefficients and 95% CIs. Clustered standard errors were computed for all models to allow for intra-center correlation.^28^, ^29^

Clinically relevant factors were selected as candidate explanatory variables in the models. For the models focusing on the first year of life, variables were assigned per patient (clinical diagnosis, gender, preterm birth, congenital noncardiac comorbidity, documented antenatal diagnosis, additional cardiac risk factor at any time during first year) and for first procedure only (age, low weight < 2.5 kg, acquired comorbidity, severity of illness marker). For the models focusing on the post-Fontan period, variables were assigned at patient level (clinical subgroup, subtype of stage 1, gender, preterm birth, congenital noncardiac comorbidity, ICU stay during first year of life, days at home during first year of life), at the time of the Fontan procedure (age, weight, weight-for-age Z score <–2, acquired comorbidity, crossclamp time), and at any record before the Fontan procedure (acquired comorbidity, severity of illness marker, total CPB in first year of life). The adjusted models include all candidate variables for the models focusing on the first year of life and those with a univariable *P* .1 or less for the models on post-Fontan procedure period, given that there were few significant variables in the latter.

All statistical analyses were performed using STATA/SE 17.0 (StataCorp LLC), and figures created using R version 4.1.2 (R Foundation for Statistical Computing).

### Stepwise Algorithm to Identify HLHS and Non-HLHS Patients

All diagnosis codes are European Pediatric Cardiac Codes.

HLHS patients.^6^

Step 1: Identify HLHS patients.

Patients with at least 1 of the following diagnosis codes.

01.01.09: HLHS.

09.15.03: Aortic atresia.

06.02.01: Mitral atresia.

07.08.42: Ventricular imbalance: dominant right ventricle and hypoplastic left ventricle (HLV).

07.07.00: Left ventricular hypoplasia.

OR procedure codes consistent with stage 1 procedure for HLHS.

12.10.00: Norwood type procedure.

12.09.03: Damus-Kaye-Stansel type procedure: pulmonary trunk to aorta end/side anastomosis AND at least 1 of the following procedure codes indicating Arterial Shunt.

12.31.03: Modified R Blalock shunt.

12.31.04: Modified L Blalock shunt.

12.31.06: Central systemic-pulmonary artery interposition shunt.

12.31.46: Modified Blalock shunt.

12.31.30: Systemic-to-pulmonary arterial shunt procedure.

OR procedure codes consistent with stage 1 or stage 2 hybrid for HLHS.

12.14.19: Application of right and left pulmonary arterial bands and 12.10.14: stent placement in arterial duct within 4 weeks.

12.10.04: Application of bilateral pulmonary arterial bands and transcatheter placement of stent in arterial duct.

12.20.20: HLHS hybrid approach (transcatheter and surgery): stage 1.

12.20.21: HLHS hybrid strategy (transcatheter and surgery)

12.20.22: HLHS hybrid approach (transcatheter and surgery) 'stage 2': aortopulmonary amalgamation + superior cavopulmonary anastomosis(es) + debanding of pulmonary arteries.

12.20.23: HLHS hybrid approach (transcatheter and surgery) 'stage 2': aortopulmonary amalgamation + superior cavopulmonary anastomosis(es) + debanding of pulmonary arteries + arch repair.

AND no diagnostic codes indicative of HLHS-related malformations from Table E1.

Step 2: Exclusion by treatment pathway not consistent with HLHS.

Removal of patients with only noncontributory or noncardiac procedures.

Removal of alive patients who had no stage 1 for HLHS by age 3 months (allow patients who had pre-pathway then died)

Removal of BV-like patients who have only pre-pathway with at least 1 of the codes 123200, 120,108, 120,103 and 120,102. Removal also patients who have pre-pathway with at least 1 of the codes 120108, 120,103 and 120,102 and a stage 1/hybrid (patients who have 123,200 pre-pathway and stage 1 will NOT be excluded).

Removal of patients with only pre-pathway and had coarctation procedures (in presence of code 121800, 121,801, 121,802, 121,803, 121,810 and 121,830).

Removal of patients who are selected by diagnosis code 070700 only and have no SV procedure and no HLHS procedure.

Removal of patients who have implausible treatment sequence or too young age at stage 2 (<2 months) or stage 3 (<6 months).

Non-HLHS patients.

These were identified in 2 stages: functionally univentricular heart (FUH) patients and then other single ventricle patients (other SV)

FUH patients.^2^

Step 1: Identify HLHS-related malformation patients.

Patients with at least 1 of the following diagnosis codes:

01.01.09: HLHS.

09.15.03: Aortic atresia.

06.02.01: Mitral atresia.

07.08.42: Ventricular imbalance: dominant right ventricle and HLV.

07.07.00: Left ventricular hypoplasia.

AND at least 1 diagnostic codes indicative of HLHS-related malformations from Table E1.

Step 2: Identify FUH patients.

Removal of any HLHS patients identified in Step 1 of HLHS.

Inclusion of HLHS-related malformation patients identified in Step 1 of FUH.

Inclusion of patients with at least 1 of the diagnosis codes from Table E2.

Include patients with SV procedures AND at least 1 of the following diagnosis codes:

Atrial isomerism (presence of codes 030104, 030,105)

AVSD (presence of codes 060726, 060,709, 060,609)

CCTGA (presence of code 010103)

Step 3: Identify and exclude patients misallocated to the FUH group.

Exclusion of patients with at least 1 of the FUH exclusion diagnostic codes from Table E3.

Step 4: Exclusion by treatment pathway not consistent with FUH.

Removal of patients with only noncontributory or noncardiac procedures.

Removal of patients who have implausible treatment sequence or too young age at stage 2 (<2 months) or stage 3 (<6 months).

Other SV patients.

Step 1: Identify and include any remaining other SV patients if

Primary diagnosis as FUH/HLHS.

OR

Had SV procedure.

Step 2: Identify and include any remaining patients who had.

A Glenn operation: 123,111, 123,115, 123,144, 123,145 or 123,172 AND at least 1 of the following diagnosis code.

Pulmonary Atresia: 010,107, 010,106, 090,512, 090,511 or 010,125.

Types of TGA: 010,118 or 010,501.

CCTGA: 010,103.

Ebstein's malformation of tricuspid valve: 060,134.

DORV: 010,104, 010,117, 010,119 or 010,140.

AND do not have the following procedure code.

Ebstein repair: 120,209.

Pulmonary valve or RVOTO opening: 120618,120,605, 120,600.

Atrial septal defect (ASD) secundum closure with transluminal device: 120,106.

Right ventricular outflow tract obstruction relief: 120,641.

Ventricular septation procedure: 120,901.

1.5 ventricle repair: superior cavopulmonary (Glenn) anastomosis + right ventricular outflow tract reconstruction: 120,619.

Step 3: Exclusion by treatment pathway not consistent with other SV.

Removal of patients with only noncontributory or noncardiac procedures.

Removal of patients who have implausible treatment sequence or too young age at stage 2 (<2 months) or stage 3 (<6 months).
